# Porphyrin-Based Covalent Organic Frameworks with Donor-Acceptor Structure for Enhanced Peroxidase-like Activity as a Colorimetric Biosensing Platform

**DOI:** 10.3390/bios13020188

**Published:** 2023-01-26

**Authors:** Qian Wang, Liang Lv, Wenhao Chi, Yujiao Bai, Wenqing Gao, Peihua Zhu, Jinghua Yu

**Affiliations:** 1 School of Chemistry and Chemical Engineering, University of Jinan, Jinan 250022, China; 2 Jinan Agricultural Product Quality and Safety Center, Jinan 250316, China

**Keywords:** porphyrin, covalent organic framework, peroxidase-like activity, cells, H_2_O_2_

## Abstract

Hydrogen peroxide (H_2_O_2_) and glucose play a key role in many cellular signaling pathways. The efficient and accurate in situ detection of H_2_O_2_ released from living cells has attracted extensive research interests. Herein, a new porphyrin-based porous covalent organic framework (TAP-COF) was fabricated via one-step condensation of 1,6,7,12-tetrachloroperylene tetracarboxylic acid dianhydride and 5,10,15,20-tetrakis (4-aminophenyl)porphyrin iron(III). The obtained TAP-COF has high surface areas, abundant surface catalytic active sites, and highly effective electron transport due to its precisely controllable donor–acceptor arrangement and 3D porous structure. Then, the new TAP-COF exhibited excellent peroxidase-like catalytic activity, which could effectively catalyze oxidation of the substrate 3,3′,5,5′-tetramethylbenzidine by H_2_O_2_ to produce a typical blue-colored reaction. On this basis, simple, rapid and selective colorimetric methods for in situ H_2_O_2_ detection were developed with the detection limit of 2.6 nM in the wide range of 0.01 to 200 μM. The colorimetric approach also could be used for in situ detection of H_2_O_2_ released from living MCF-7 cells. This portable sensor based on a COF nanozyme not only opens a new path for point-of-care testing, but also has potential applications in the field of cell biology and clinical diagnosis.

## 1. Introduction

Small molecule biomarkers are a significant part of sustaining life processes and biological metabolism [1]. Among them, hydrogen peroxide (H_2_O_2_), one of the frequent reactive oxygen species in biological systems, is essential for promoting cellular proliferation, differentiation, and emigration [2,3]. Various illnesses including cancer [4], diabetes mellitus [5], neurological disorders [6], and angiocardiopathy [7] are tightly accompanied by abnormal H_2_O_2_ production. For example, cancer cells have considerably greater amounts of H_2_O_2_ (6.0 μM–1.2 mM) than normal cells (less than 0.72 μM) [8]. Therefore, the need for a reliable and accurate approach for the extremely hypersensitive identification of H_2_O_2_ in living creatures is critical. The examination of H_2_O_2_ has been reported using a variety of strategies, including electrochemical [9], fluorescence [10,11], electrochemiluminescence [12], spectrophotometry [13], etc., among which colorimetric tests [14] are considered to be an effective and straightforward method due to its affordability and usefulness.

Peroxidases, like horseradish peroxidase (HRP) and thyroid peroxidase, are found in plankton and flora and fauna, which are commonly utilized because of their high particularity and effectiveness under mild settings [15]. However, due to their inherent nature as proteins, the majority of natural enzymes have many inevitable shortcomings, such as intrinsic instability, costly preparation, and easy denaturation under adverse conditions, which severely restricts their real applications [16]. Then, a lot of work has been put into researching nanozymes, such as porphyrins and metal complexes, with the aim of simulating the structures and activities of naturally produced enzymes. Due to their affordability, a large-scale production capacity, superior stability and robustness compared to natural enzymes, the interests in nanozymes with unexpected enzyme-like activity have grown recently.

Porphyrin-based nanozymes are currently receiving more focus for their biological similarity to inartificial enzymes like cytochrome P450 and hemoglobin [17]. Various iron porphyrins, including hemin [18] and 5,10,15,20-tetrakis-(4′-bromophenyl)porphyrinato iron(III) [19] are used to simulate HRP activity. Free iron porphyrins cannot, however, be used in standard immunoassay platforms because of their poorer imitation of peroxidase activity and facile aggregation property [20]. Recently, a lot of interest has been focused on porphyrin-based covalent organic frameworks (COFs), which are produced from porphyrin or metalloporphyrin through strong covalent bonding, as biomimetic catalysts, electrocatalysts, and photocatalysts [21,22,23] due to their superior catalytic operations and close biological affinity to inartificial enzymes. These possess COFs’ intrinsic benefits, including their high surface area, tunable pore topologies, functionalization, and excellent thermal and chemical durability. By incorporating metalloporphyrin into COFs, high density active sites would be added to the skeleton of the COFs, and the catalytic deactivation brought on by porphyrin aggregation might also be prevented. Typically, Song’s group proposed a series of ultrastable iron porphyrin-based COFs with 3D channels as biomimetic catalysts [24]. Lin’s group created a 3D porphyrin-based COFs through the co-condensation of tetrahedrons with parallelogram construction blocks to enhance catalytic performance [25]. Additionally, to produce small band gaps for the dissociation and transportation of electron-hole pairs, the appropriate combination and arrangement of complementary donor–acceptor pairs inside the COFs skeleton have been shown to be an effective strategy to generate higher catalytic activity. Meanwhile, the unitarity of the COFs would be advantageous to boost the efficiency of enzymatic processes by improving the stable enzymes and decreasing the diffusion barrier.

Inspired by the above discussion, a donor–acceptor COFs (defined as TAP-COF) with 5,10,15,20-tetrakis (4-aminophenyl)porphyrine iron(III) chloride (FeTAPP) as the donor and 1,6,7,12-tetrachloroperylene tetracarboxylic acid dianhydride (TAD) as the acceptor was synthesized and employed as a peroxidase-mimicking nanozyme. The produced D-A COFs had a high degree of crystallinity, a sizable amount of porosity, excellent electron transport, and a significant number of catalytic active sites. Moreover, TAP-COF was a metalloporphyrin-based COFs with a built-in catalyst that could successfully prevent deactivation, which was frequently linked to porphyrin aggregation. TAP-COF was utilized to build a colorimetric assay in viable cells for in situ analysis of H_2_O_2_ using of 3,3′,5,5′-tetramethylbenzidine (TMB), which offered a novel method of cell monitoring and has promising uses in the field of fast clinical detection.

## 2. Materials and Methods

### 2.1. Peroxidase-like Performance of TAP-COF

By catalytically oxidizing the peroxidase zymolyte TMB in the absence of H_2_O_2_, the peroxidase-like activity of TAP-COF was examined. The measurements were made by monitoring the absorbance information in time-scan patterns at 652 nm. In the typical experiment, 200 μL 0.32 mg·mL^−1^ TAP-COF dispersed in water, 200 μL H_2_O_2_ (30 mM), and 200 μL TMB (0.80 mM) as the substrate were added to a HAc–NaAc buffer solution (pH 3.8).

In a typical colorimetric experiment, 200 μL TAP-COF (0.32 mg·mL^−1^) dispersed in water, 200 μL H_2_O_2_ (30 mM), and 200 μL *tert*-butyl alcohol (1 mM) were added into 1200 μL of a HAc–NaAc buffer solution (pH 3.8). Next, the above solution was incubated at 25 °C for 0.5 h and 200 µL TMB (0.80 mM) was added before incubating for another 10 min. Subsequently, UV–vis absorption spectroscopies were used to gauge the final reaction solution. In comparison, the same experimental conditions were used to investigate the expression system of TAP-COF + H_2_O_2_ + TMB + buffer by omitting the *tert*-butyl alcohol.

### 2.2. Condition Optimization

The influences of the pH values (1.7–8.0), temperatures (25–70 °C), and various concentrations of H_2_O_2_ (1–400 mM) and TMB (0.20–2.5 mM) were investigated to evaluate the peroxidase-like activity of the TAP-COF composites. The effect of the catalyst amount was also studied.

### 2.3. Detection of H_2_O_2_ in Cells

A total of 200 μL phorbol 12-myristate-13-acetate (PMA) (50 g·mL^−1^) was injected to a counting plate containing 0, 100, 200, 500, 1000, 3000, or 5000 cells·mL^−1^ MCF-7 cells in order to determine the amount of H_2_O_2_ released by the cells. To facilitate color development, 200 μL TMB (0.8 mM) and 200 μL TAP-COF (0.32 mg·mL^−1^) were injected to each counting plate after the specimens had been incubated for 10 min in 0.2 M HAc–NaAc (pH 4.0). A microplate reader was used to measure each well’s absorbance at 652 nm, which was believed to be related to the emission of H_2_O_2_ in cells.

## 3. Results and Discussions

### 3.1. Structure and Morphology Characterization

**Scheme 1:**
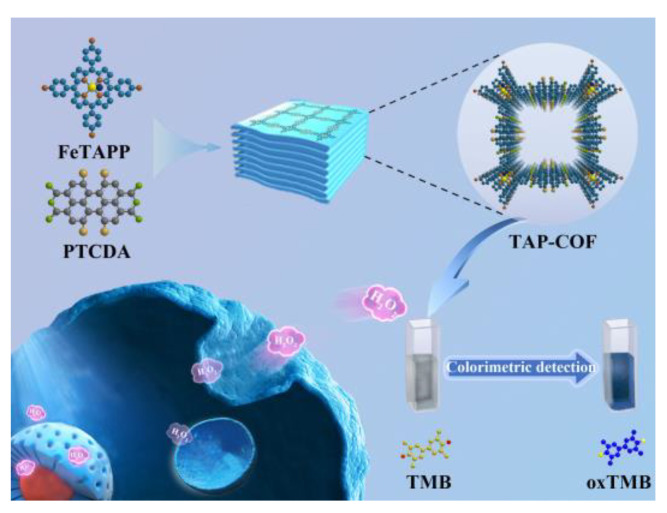
Diagrammatic drawing for the synthesis of TAP-COF and in situ detection of H_2_O_2_ released from MCF-7 cells.

**Figure 1:**
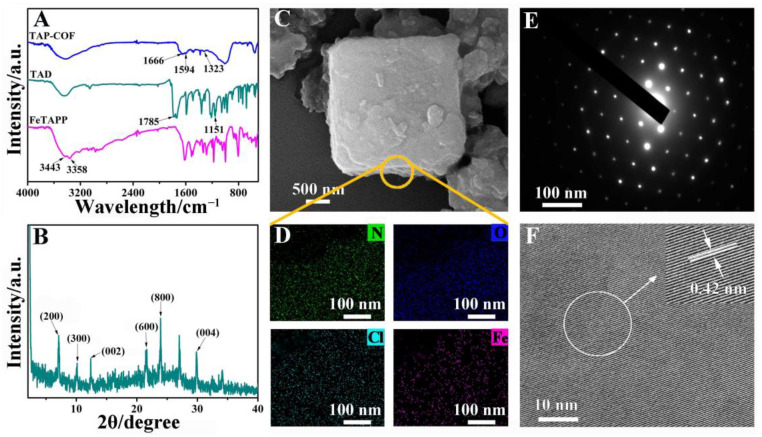
(**A**) FT-IR spectra of FeTAPP, TAD, and TAP-COF; (**B**) XRD spectra of the TAP-COF; (**C**) SEM image; (**D**) N, O, Cl, Fe element mapping; (**E**) SAED pattern from the crystalline domain; (**F**) HRTEM image of TAP-COF.

TAP-COF was synthesized by condensation between FeTAPP with TAD in the presence of 4 Å molecular sieves under solvothermal conditions (Scheme 1). To confirm the formation of the diimide linkages between FeTAPP and TAD units in the TAP-COF, Fourier transform infrared (FT-IR) spectroscopy was performed (Figure 1A). FT-IR spectra indicated that the peak of the N-H stretching of -NH_2_ (at 3364 cm^−1^) from FeTAPP disappeared after polymerization. Meanwhile, the C = O amide stretching vibrations correspond to the absorptions at 1666 cm^−1^. The peak at 1323 cm^−1^ was attributed to the C-H in phenyl rings’ in-plane bending vibration. In addition, with the presence of C-N-C, a clear band was displayed at 1594 cm^−1^ [26]. However, the peaks belonging to anhydride were not accurately identified due to the synthesis of imide between amino groups and anhydride. The X-ray diffraction (XRD) measurement indicated that TAP-COF was a well crystalline material as illustrated in Figure 1B. The XRD diagram of TAP-COF demonstrated two peaks at 1.26 nm and 0.72 nm, which were ascribed to diffraction from the (200) and (002) planes, respectively. In addition, the XRD pattern also displayed three higher order refractions at 0.88, 0.42, and 0.38 nm, which were ascribed to diffraction from the (300), (600), and (800) planes, respectively, revealing the high molecular ordering nature of this COF along the a-axis of the unit cell [27]. The (001) plane gave its higher order diffractions at 0.34 nm in the wide-angle range of the XRD pattern. These diffraction results could be assigned to refraction from a rectangular lattice with the cell parameter of a = 2.52 nm, b = 2.52 nm, and c = 1.44 nm (Figure S1 in Supplementary Materials). In addition, the XRD pattern of TAP-COF exhibited additional refraction at 0.34 nm, which was attributed to the π-π stacking distance between (100) planes [28,29,30].

The morphologies of TAP-COF were analyzed using scanning electron microscopy (SEM). The SEM image (Figure 1C) revealed that TAP-COF had a distinct lamellar structure. In addition, the energy-dispersive spectrometry mapping was exhibited in Figure 1D. Atoms of the expected composition (N, O, Cl, Fe) of the compound could be detected, suggesting that TAP-COF was synthesized successfully. In order to further investigate the morphology of TAP-COF, high-resolution transmission electron microscopy (HRTEM) was also performed. The sample’s crystalline nature was confirmed by the regular spot pattern that was revealed by the selected area electron diffraction (SAED) in the chosen location, as exhibited in Figure 1E. The HRTEM image indicated that the lattice space of the TAP-COF was calculated to be 0.42 nm (Figure 1F), which corresponded to (600) plane of TAP-COF as seen in the XRD profile.

To further confirm the thermal stability of TAP-COF, thermal gravimetric analysis (TGA) under air conditions of the sample was conducted. As illustrated in Figure S2, the TGA presented a rapid weight loss of ~12.2% in the temperature range of 25–150 °C, which seemed to be ascribed to the loss of the solvent absorbed in the porous sample [31]. Then, the TGA profile of the TAP-COF did not exhibit any weight loss in the temperature range of 150–380 °C. In addition, TAP-COF showed an observable weight loss over 380 °C, which was attributed to the decomposition of the frameworks. Based on the above analysis, the TGA curves revealed that the TAP-COF was thermally stable up to 380 °C.

The permanent porosity of TAP-COF was checked by N_2_ adsorption isotherms collected at 77 K (Figure S3). The sorption isotherm fit well with the type IV characters, suggesting the permanent mesoporous nature of TAP-COF. According to the Figure S3, the COF had a total pore volume of 0.124 cm^3^·g^−1^ (P/P_0_ = 0.99). In addition, the BET and Langmuir surface areas of TAP-COF were calculated to be 66.38 and 140.12 m^2^·g^−1^, respectively, which indicated the small surface area of TAP-COF compared to other COFs. It might be related to the disorganized stacking of the TAP-COF layers caused by the steric hindrance of TAD and the twisting configuration of the phenyl units in FeTAPP.

### 3.2. Peroxidase-like Activity of TAP-COF

**Figure 2:**
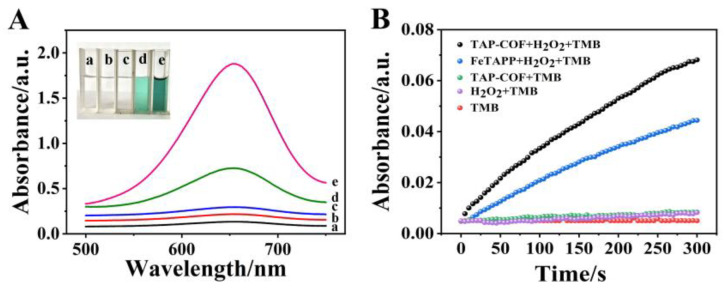
The absorbance changes of TMB, H_2_O_2_ + TMB, TAP-COF + TMB, FeTAPP + H_2_O_2_ + TMB, and TAP-COF + H_2_O_2_ + TMB are illustrated in (**A**) UV–vis spectra and (**B**) the impact of time on these changes. TMB reaction mixture’s color variations are seen in the inset.

The peroxide simulation properties of TAP-COF were examined by oxidizing the colorimetric primer TMB to oxTMB, which could be easily monitored by the UV–vis absorbance spectroscopy or observed by the naked eyes. Figure 2A shows the UV–vis absorption spectra for the different test solutions. The absence experiments were performed to determine the components needed to trigger the reaction. When both TAP-COF and H_2_O_2_ were present in the reaction system, there was a higher absorption peak at 652 nm, indicating that TAP-COF and H_2_O_2_ were required for the catalytic process. Additionally, Figure 2B shows the variations of absorbance over time for TAP-COF + H_2_O_2_ + TMB and H_2_O_2_ + TMB + FeTAPP. The absorbance at 652 nm rose for each system as the reaction proceeded, but the TAP-COF + H_2_O_2_ + TMB system responded much more quickly than the other systems. After only five minutes of reaction, the absorbance of TAP-COF + H_2_O_2_ + TMB exceeded that of FeTAPP + H_2_O_2_ + TMB, indicating that TAP-COF had a greater catalytic activity than FeTAPP. These findings unequivocally indicated that TAP-COF had a better sensitivity for accelerating the oxidation of TMB. The inherent skeleton structure, high N content, and D-A structure of TAP-COF might be the origin of its porosity characteristics, electron cloud density, and greater surface catalytic activity sites. These would hasten the electron exchange between reactive oxygen species generated by catalyzing hydrogen peroxide decomposition and TMB, thus increasing the catalytic activity. They also had electrostatic interaction that bind the catalytically active sites of TAP-COF to the positively charged TMB.

### 3.3. Optimization of Experimental Conditions

Similar to HRP and other nanomaterial-based peroxidase mimics, the pH, temperature, and substrate concentration during the color development process also influenced the catalytic performance of TAP-COF. Then, the peroxidase-like activity of TAP-COF was investigated by varying the pH (1.7–8), temperature (25–70 °C), H_2_O_2_ concentration (0–200 M), and TMB concentration (0.2–2.5 mM) as shown in Figure S4. These data suggested that the ideal experimental settings were pH 3.8, 25 °C, the relative lowest necessary concentration of H_2_O_2_ (200 μM) and 0.8 nM of TMB. Moreover, the catalytic activity of TAP-COF was also related to its amount. The catalytic activity increased with a rapid increase in catalyst concentration from 0 to 1.0 mg·mL^−1^ and then there was a downtrend from 1.0 to 1.5 mg·mL^−1^. Thus, the optimal concentration of TAP-COF was chosen as 1.0 mg·mL^−1^.

### 3.4. Steady-State Kinetic Analysis

Typical Michaelis–Menten curves were produced under ideal conditions over a range of TMB or H_2_O_2_ concentrations to quantitatively explore the catalytic activities of the TAP-COF (Figure S5). Under the guidance of the Lineweaver–Burk equation, the Michaelis constant (K_m_) and the maximum response velocity (V_max_) were calculated (Table 1). In general, K_m_ describes how well an enzyme binds to its substrates, with a lower K_m_ value indicating a stronger binding. The K_m_ of TAP-COF (0.78 mM) for H_2_O_2_ as primer was significantly lower than that of HRP (3.7 mM), demonstrating that the affinity of TAP-COF for H_2_O_2_ was stronger than that of HRP. This might be because of the increased electrostatic contact between TMB and TAP-COF produced by its high N content. Furthermore, the TAP-COF had a K_m_ of 0.18 mM with TMB as the substrate, which was lower than the HRP’s K_m_ of 0.43 mM, indicating that TAP-COF also had a high affinity for the TMB mediator. These results implied that TAP-COF had promising peroxidase-like activity, which was in line with other discoveries using various peroxidase mimics based on nanomaterials (Table 1).

### 3.5. Colorimetric Sensing

**Figure 3:**
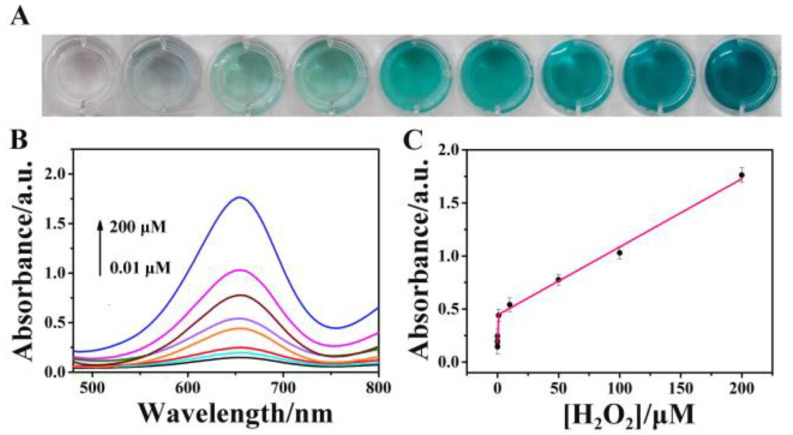
Photographs (**A**) of the TMB + H_2_O_2_ colorimetric reaction solutions facilitated by various H_2_O_2_ concentrations in the range of 0.01–200 μM and (**B**) UV–vis absorption spectra. (**C**) Relationship between absorbance at 650 nm in reaction solutions’ UV–vis absorption spectra and H_2_O_2_ concentration. Standard deviations are shown by error bars (*n* = 5).

The catalysis-based colorimetric test revealed extremely sensitive changes in absorption with varying H_2_O_2_ concentrations. Figure 3A demonstrates that the immunoassay solutions gradually darkened from colorless to blue as the H_2_O_2_ content increased. Additionally, Figure 3B and C illustrates a superb linear correlation between the logarithm of H_2_O_2_ concentration in the range of 0.01 μM to 200 μM and the absorbance of oxidized TMB at 652 nm. The TAP-COF-based sensor had a lower limit of detection (LOD) and larger linear range compared to other previously published outstanding peroxidase-based sensors (see Table S1). Additionally, our proposed sensor’s LOD was lower than that of some of the other electrochemical and fluorescent sensors, which might be explained by the fact that the interlinking of electron acceptor and donor of TAP-COF could improve the electron transport rate, as well as allow exposure to high concentrations of catalytic active sites, thus contributing to its increased catalytic activity.

**Figure 4:**
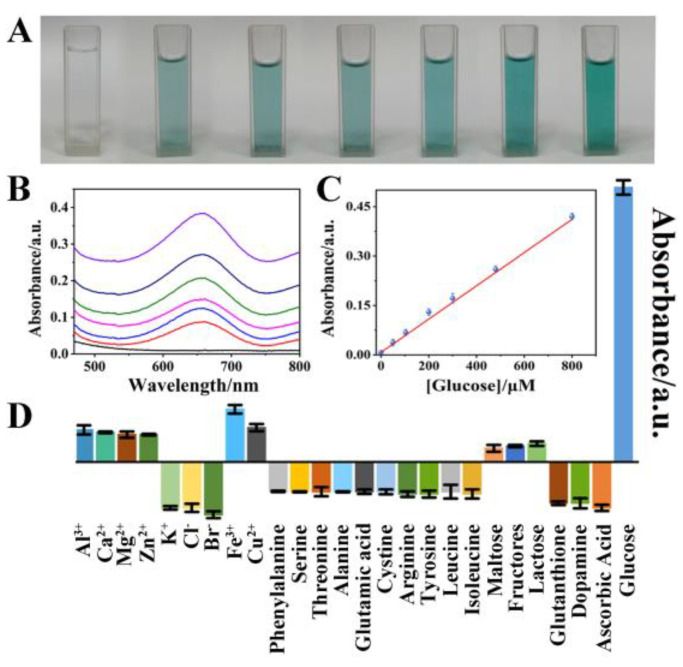
(**A**) A picture of the reaction mixture with various concentrations of glucose present. (**B**) The UV–vis absorption spectra. (**C**) Linear calibration of glucose detection. (**D**) The absorption of TMB-TAP-COF for different substances. (The concentration of potentially interfering substances was 50 μM, and the concentration of glucose was 0.5 μM, TMB was 0.8 mM and TAP-COF was 1.0 mg·mL^−1^).

Moreover, H_2_O_2_ was also a major byproduct of other metabolic processes, including the oxidation of uric acid by the enzyme uricase as well as the oxidation of glucose by the enzyme glucose oxidase (GOD). Consequently, the measurement of H_2_O_2_ might be used to indirectly infer the quantities of living components. Figure 4A displays the TMB + TAP-COF + GOD photographs at various glucose concentrations. As the glucose concentration increased linearly, the reaction became progressively darker blue in the presence of TAP-COF and GOD. The standard curves for these reactions are shown in Figure 4C, where the linear range of glucose was 0.3–800 μM, and the LOD was 0.14 μM, which was comparable to that of other reported fluorescent probes (Table S2).

### 3.6. Selectivity for the Detection of Glucose

Several interference experimental studies were conducted in the presence of interfering chemicals to determine the selectivity of TAP-COF-based colorimetric sensors for H_2_O_2_ detection (Figure 4D). The response of glucose (2 × 10^−4^ mmol·L^−1^) was up to 100 times greater than that of other substances (2 × 10^−2^ mol·L^−1^), none of which interfered with the measurement of glucose. These outcomes demonstrated the TAP-COF-based sensor exhibited superior glucose detection selectivity.

### 3.7. Colorimetric Detection Mechanism for H_2_O_2_

**Figure 5:**
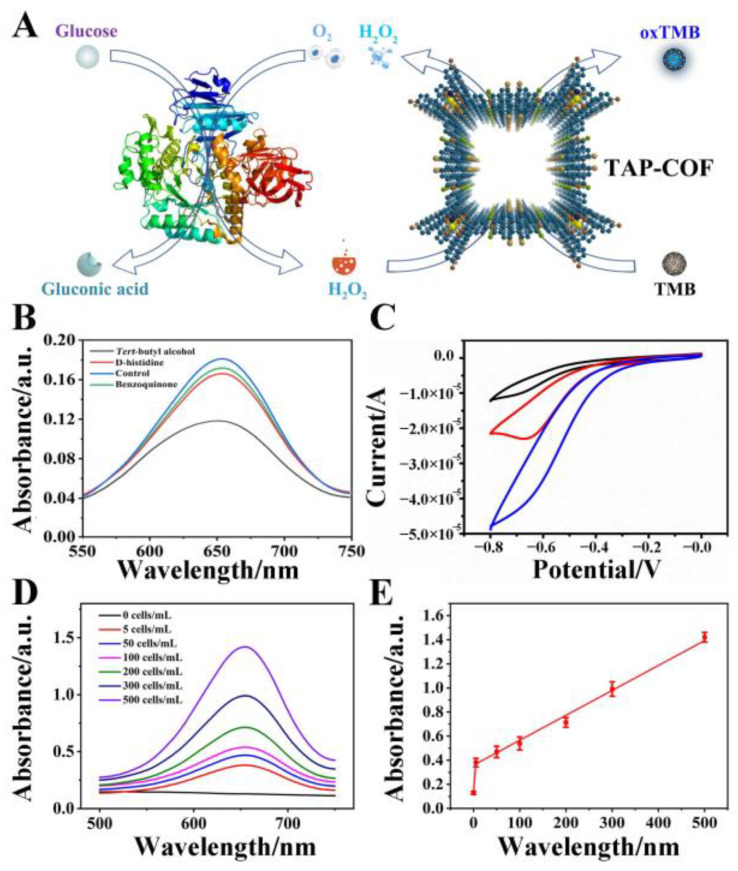
(**A**) Diagrammatic representation of the blue-colored oxTMB produced in cells by the INAzyme-catalyzed cascade oxidation of glucose and TMB. (**B**) ROS trapping experiment. The TMB oxidation process in the presence of D-histidine (^1^O_2_ scavenger), tert-butyl alcohol (**·**OH scavenger), and benzoquinone (O_2_^•−^ scavenger). (**C**) CV curves of different electrodes, GCE electrode (black line), FeTAPP/GCE (red line), TAP-COF/GCE (blue line). TAP-COF-based colorimetric examination of MCF-7 cells in binding buffer for quantitative analysis of (**D**) UV-vis absorption spectroscopy and (**E**) linear calibration of MCF-7 cells detection.

The cascade reaction-based colorimetric glucose assay is depicted in Figure 5A. The peroxidase-like activity of TAP-COF was derived from its catalytic H_2_O_2_ decomposition to produce ROS, mainly including singlet oxygen (^1^O_2_), hydroxyl radical (**·**OH), and superoxide anion (O_2_**^•^**^−^), where **·**OH was a possible source of the catalytic process of TMB [36,37]. Next, capture experiments were used to test the ROS, and the catalytic mechanism and specificity of TAP-COF was further studied. As demonstrated in Figure 5B, the TMB + H_2_O_2_ + TAP-COF system showed different absorbance intensity with or without the addition of D-histidine (^1^O_2_ scavenger), *tert*-butyl alcohol (**·**OH scavenger), and benzoquinone (O_2_**^•^**^−^ scavenger). The absorbance significantly decreased after the addition of *tert*-butyl, suggesting that the generated **·**OH was scavenged by *tert*-butyl alcohol. Meanwhile, the system’s absorbance value exhibited no evident change after the addition of D-histidine and benzoquinone, indicating the specificity of TAP-COF peroxide-mimicking enzymes [38]. In summary, the peroxide-mimicking enzyme activity of TAP-COF mostly resulted from the **·**OH, which was at odds with earlier studies on certain inorganic nanomaterials that were thought to originate via electron transport [39]. The electrocatalytic behavior of the TAP-COF and the FeTAPP modified GCE towards the electrochemical reduction of H_2_O_2_ was examined in further detail utilizing amperometric responses. Compared with the FeTAPP-modified electrode, the TAP-COF-modified electrode exhibited an enhanced reduction peak current, suggesting that TAP-COF exhibited higher electrocatalytic activity in the reduction of H_2_O_2_, which indicated that the TAP-COF had the better ability to accelerate the electron transfer (Figure 5C). Based on the above discussion, the possible explanation of TMB substrate oxidation by the TAP-COF catalyst in the presence of H_2_O_2_ might be proposed as follows: first, H_2_O_2_ entered the pores of TAP-COF or attached to the surface, and then the O–O bond of H_2_O_2_ broke to form **·**OH. Second, **·**OH oxidized TMB to produce a blue-colored byproduct. These phenomena indicated that the blue signal came from the charge transfer complex (chromogen). Electrochemical studies have indicated that there was a high degree of communication between the donor and acceptor of TAP-COF and the entire molecule’s electrical activity was governed by this connection. Moreover, the catalytic oxidation of TMB by H_2_O_2_ was aided by the D-A ordered structure of TAP-COF’s capacity to speed electron transport. Therefore, the design of TAP-COF with D-A properties was of great significance for the construction of novel colorimetric sensors.

### 3.8. Colorimetric Detection of Cancer Cells

H_2_O_2_ was an important component of several metabolic processes, including the metabolism of proteins and carbohydrates, and it may be used as a marker for a number of serious diseases, including cancer [40]. Then, samples with various MCF-7 cells ranging from 0–3000 cells·mL^−1^ were identified in 100 μL serial dilutions of binding buffer using TAP-COF as described above. Without including MCF-7 cells, the absorbance of the background was estimated using a similar approach (Figure 5D), and the absorbance at 650 nm was gathered for profiling. As demonstrated in Figure 5E, with the minimum cell concentration of 25 cells·mL^−1^ observed in our actual studies, a strong linear correlation was displayed between the colorimetric signal and the cell concentration (R^2^ > 0.98). This LOD was significantly lower than that attained using the currently available colorimetric cancer cell detection techniques, and even on par with that attained using electrochemical or fluorescent assays that are more sensitive [41,42,43]. The above results indicated that the TAP-COF-based colorimetric biosensor displayed a powerful ability to detect H_2_O_2_ released from cancer cells, and thus our proposed simple and convenient colorimetric assay could be used for the early diagnosis of cancer cells.

## 4. Conclusions

In conclusion, a new functional TAP-COF nanozyme with a donor–acceptor arrangement has been constructed through one-step condensation. The TAP-COF demonstrated increased enzyme mimicking activities for the direct catalysis of TMB to create blue oxTMB due to very efficient charge transfer, porous strutted structure, and the abundance of iron centers. Correspondingly, an H_2_O_2_-sensitive colorimetric detection method based on the TAP-COF sensing system was established by facilitating the electron change between H_2_O_2_ and TMB. The established colorimetric biosensor has high selectivity, excellent compatibility, and outstanding sensitivity, which was successfully used for the visual detection of H_2_O_2_ released from living cells. Our work provides a portable COF nanozyme-based sensing platform for the accurate detection of small molecule biomarkers, and a new perspective for point-of-care testing and medical diagnosis.

## Figures and Tables

**Table 1:** Comparison of maximal reaction rate (V_max_) and apparent Michaelis-Menten constant (K_m_).

Catalyst	K_m_/mM		V_max_/10^−8^ M s^−1^		Reference
	H_2_O_2_	TMB	H_2_O_2_	TMB	
FePor-TFPA-COP	2.77	0.027	83.3	1.76	[32]
H_2_TCPP-Co_3_O_4_	3.70	0.43	8.71	10.0	[33]
Fe-MIL-88NH_2_	6.10	0.028	0.710	0.670	[34]
C-Dots	39.1	0.011	1.40	4.80	[35]
TAP-COF	0.780	0.18	2.94	4.78	This work

## Data Availability

Not applicable.

## Supplementary Materials

The following supporting information can be downloaded at: https://www.mdpi.com/article/10.3390/bios13020188/s1. Figure S1: Schematic representation of the unit cell in the TAP-COF (A) top view and (B) side view; Figure S2: TGA data of TAP-COF; Figure S3: N2 adsorption-desorption isotherm curve of TAP-COF at 77 K; Figure S4: Exploring the impacts of this detection system, including (A) temperature, (B) pH, (C) TMB concentration, (D) COF concentration, and (E) H2O2 concentration; Figure S5: Steady-state kinetic analysis using the Michaelis-Menten model (A, B) and Lineweaver-Burk model (C, D) for TAP-COF. The concentration of H2O2 was 60 mM and TMB concentration was varied (A, C). The concentration of TMB was 0.80 mM and H2O2 concentration was varied (B, D); Table S1: Detection limit comparison of TAP-COF for H2O2 with some recently reported peroxidase mimics; Table S2: Comparison of analytical parameters for the determination of glucose. References [44,45,46,47,48,49,50,51,52,53,54,55,56,57,58,59] are cited in Supplementary Materials.
