# Supplementary material for: Porphyrin-Based Covalent Organic Frameworks with Donor-Acceptor Structure for Enhanced Peroxidase-like Activity as a Colorimetric Biosensing Platform
Source: Biosensors (Basel). 2023 Jan 26;13(2):188. doi: 10.3390/bios13020188 (PMC9953433; doi:10.3390/bios13020188)
Supplement: Supplementary file 1 [file biosensors-13-00188-s001.zip › biosensors-2173261-supplementary.pdf]

biosensors
MDPI

Supporting Information

Porphyrin-Based Covalent Organic Frameworks with Donor-Acceptor Structure for Enhanced Peroxidase-Like Activity as a Colorimetric Biosensing Platform

Qian Wang [1,†], Liang Lv [2,†], Wenhao Chi [1], Yujiao Bai [1], Wenqing Gao [1], Peihua Zhu [1,*], Jinghua Yu [1]

[1] School of Chemistry and Chemical Engineering, University of Jinan 250022, China
[2] Jinan Agricultural Product Quality and Safety Center, Jinan 250316, China
* Correspondence: chm_zhuph@ujn.edu.cn
† These authors equally contributed to this work.

1. Materials and Methods

2.1. Materials

MCF-7 cells were purchased from Sigma Aldrich and stored in a refrigerator at −18 °C. 3,3′5,5′-tetramethylbenzidine (TMB) was purchased from Shanghai Yuanye Bio-Technology Co., Ltd. (Shanghai, China). p-Phthalic acid and 1,6,7,12-tetrachloroperylene tetracarboxylic acid dianhydride were purchased from Shanghai Macklin Biochemical Co. Ltd. (Shanghai, China). All reagents were of analytical reagent grade and used as received without further treatment. All aqueous solutions were prepared using ultrapure water (Milli-Q, Millipore). The 5,10,15,20-tetrakis (4-aminophenyl)porphyrine iron(III) chloride (FeTAPP) were prepared according to the published procedure[1,2].

2.2. Characterization

Electronic absorption spectra were obtained using a Shimadzu UV-2550 UV-visible spectrophotometer. Fourier transform infrared (FT-IR) spectra were recorded as KBr pellets using a Bruker Vertex 70 infrared spectrometer with 2 cm^{-1} resolution. The morphology was observed with scanning electron microscopy (SEM, JEOL 6700-F) and high-resolution transmission electron microscopy (HRTEM, JEM-2100, JEOL, Japan). For SEM imaging, Au (1-2 nm) was sputtered onto the substrate to prevent charging effects and to improve the image clarity. For HRTEM imaging, a drop of sample solution was cast onto a carbon copper grid. The X-ray diffraction (XRD) measurements were carried out on an X-ray diffractometer (Bruker D8 Focus) with Cu $K\alpha 1$ radiation (λ = 0.15406 nm) at room temperature. Thermogravimetric analysis (TGA) experiments were performed on a Mettler Toledo TGA/SDTA851 interfaced with a PC using Star software. Samples were heated at a rate of 10 °C/min under a nitrogen atmosphere.

2.3. Synthesis of TAP-COF

In this work, the TAP-COF was synthesized according to the previous reports[3,4]. In a typical procedure, 1,6,7,12-tetrachloroperylene tetracarboxylic acid dianhydride (TAD) (106 mg, 0.20 mmol), FeTAPP (50 mg, 0.1 mmol), 4Å molecular sieves and molten imidazole in a 20 mL pyrex tube was degassed by three freeze-pump-thaw cycles. The tube was sealed and heated at 170 °C for 3 days. The resultant solid was separated by centrifugation and rinsed with distilled water, 3% NaOH, chloroform, and methanol to get rid of any unreacted starting ingredients after cooling to room temperature. The TAP-COF was dried under vacuum for 12 hours at 120 °C to produce a dark purple powder with an isolated yield of about 90%.

2.4. Kinetic Analysis

Kinetic measurements were carried out in time-drive mode by monitoring the absorbance change at 652 nm. The TMB oxidation kinetics was further studied through steady-state kinetics measurement where the experiments were performed by changing one substrate concentration while keeping the strength of other component constant. The obtained absorbance values were converted to the concentration of the blue product by applying molar attenuation coefficient value of 39000 $M^{-1}\cdot cm^{-1}$. Experiments were performed using 1 $mg\cdot mL^{-1}$ TAP-COF in 1.4 mL of reaction buffer (0.20 M acetate buffer, pH 3.8) with 0.80 mM TMB as the substrate or 60 mM H_2O_2 as the substrate, unless otherwise stated. The apparent kinetic parameters were calculated using Lineweaver-Burk plots of the double reciprocal of the

Michaelis-Menten equation: $1/\upsilon = K_m/V_{max}(1/[S] + 1/K_m)$, where υ is the initial velocity, V_{max} is the maximal reaction velocity, [S] is the concentration of substrate and K_m is the Michaelis constant.

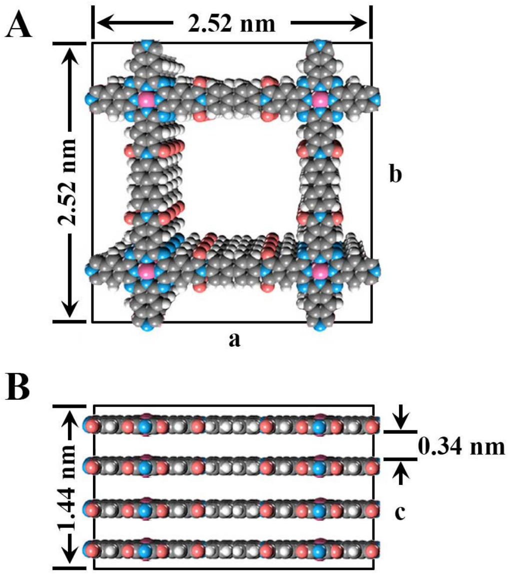

Figure S1. Schematic representation of the unit cell in the TAP-COF (A) top view and (B) side view.

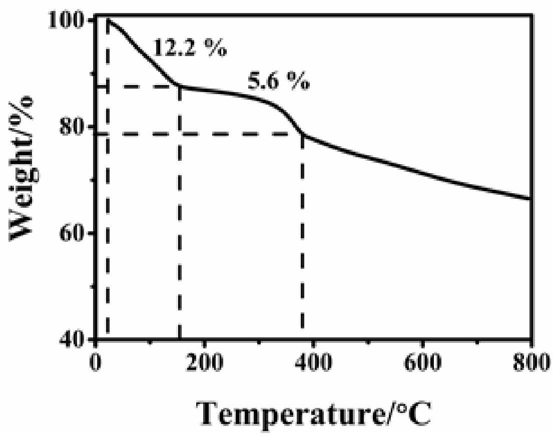

Figure S2. TGA data of TAP-COF.

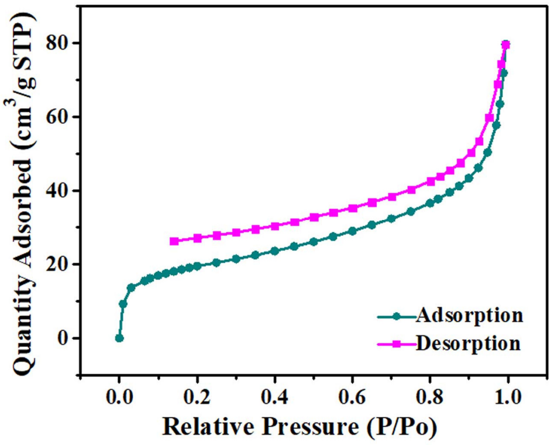

Figure S3. N_2 adsorption-desorption isotherm curve of TAP-COF at 77 K.

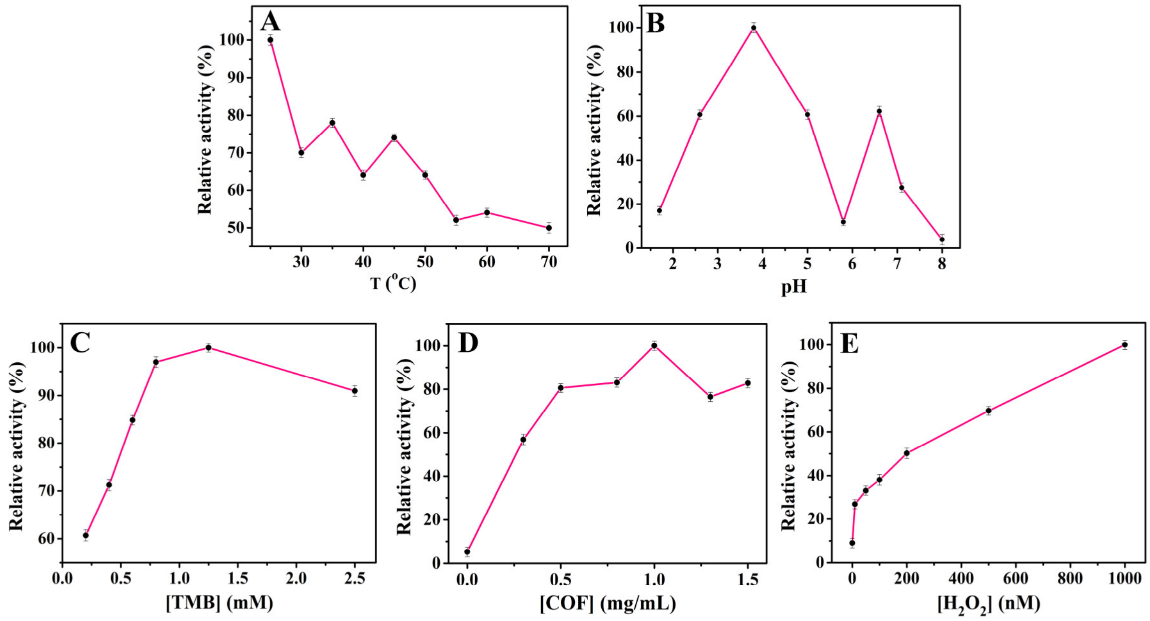

Figure S4. Exploring the impacts of this detection system, including (A) temperature, (B) pH, (C) TMB concentration, (D) COF concentration, and (E) H_2O_2 concentration.

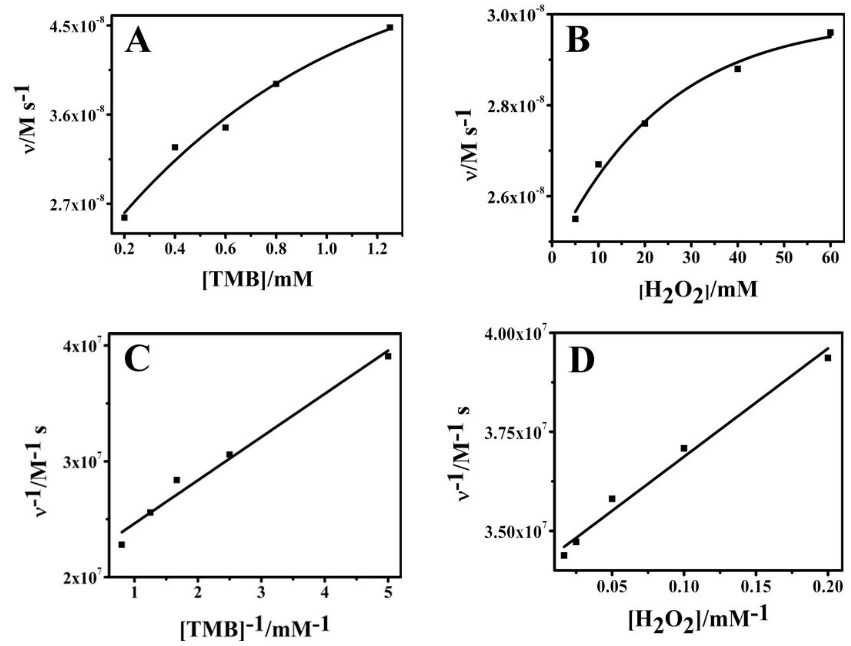

Figure S5. Steady-state kinetic analysis using the Michaelis-Menten model (A, B) and Lineweaver-Burk model (C, D) for TAP-COF. The concentration of H_2O_2 was 60 mM and TMB concentration was varied (A, C). The concentration of TMB was 0.80 mM and H_2O_2 concentration was varied (B, D).

Table S1. Detection limit comparison of TAP-COF for H_2O_2 with some recently reported peroxidase mimics.

Materials	Limit of detection (μM)	Linear range (μM)	Reference
FePPOP-1	6.5	20-1000	[5]
$Cu_2(OH)_3Cl$-CeO_2	10	20-50	[6]
GO-FeTPyP NCs	72	20-500	[7]
CoS	20	50-800	[8]
FePt-Au HNPs	12	20-700	[9]
TAP-COF	2.6×10^{-3}	0.01-200	This work

Table S2. Comparison of analytical parameters for the determination of glucose.

Methods	Limit of detection (μM)	Linear range (μM)	Reference
Fluorometry	8	10-100	[10]
	5	5×10^4-1×10^6	[11]
	760	250-8000	[12]
Electrochemistry	3	10-13000	[13]
	53.2	1×10^3-1.4×10^4	[14]
	400	5×10^4-1×10^5	[15]
Colorimetry	36	50-500	[16]
	0.15	0.3-800	This work

References

1. Abdinejad, M.; Dao, C.; Zhang, X.A.; Kraatz, H.B. Enhanced Electrocatalytic Activity of Iron Amino Porphyrins Using a Flow Cell for Reduction of CO_2 to CO. *J. Energy Chem.* **2021**, *58*, 162-169, doi:10.1016/j.jechem.2020.09.039.
2. Pegis, M.L.; Martin, D.J.; Wise, C.F.; Brezny, A.C.; Johnson, S.I.; Johnson, L.E.; Kumar, N.; Raugei, S.; Mayer, J.M. Mechanism of Catalytic O_2 Reduction by Iron Tetraphenylporphyrin. *J. Am. Chem. Soc.* **2019**, *141*, 8315-8326, doi:10.1021/jacs.9b02640.

3. Hartnett, P.E.; Mauck, C.M.; Harris, M.A.; Young, R.M.; Wu, Y.L.; Marks, T.J.; Wasielewski, M.R. Influence of Anion Delocalization on Electron Transfer in a Covalent Porphyrin Donor-Perylenediimide Dimer Acceptor System. *J. Am. Chem. Soc.* **2017**, *139*, 749-756, doi:10.1021/jacs.6b10140.
4. Zhang, C.; Zhang, S.; Yan, Y.; Xia, F.; Huang, A.; Xian, Y. Highly Fluorescent Polyimide Covalent Organic Nanosheets as Sensing Probes for the Detection of 2,4,6-Trinitrophenol. *ACS Appl. Mater. Interfaces* **2017**, *9*, 13415-13421, doi:10.1021/acsami.6b16423.
5. Cui, C.; Wang, Q.; Liu, Q.; Deng, X.; Liu, T.; Li, D.; Zhang, X. Porphyrin-Based Porous Organic Framework: An Efficient and Stable Peroxidase-Mimicking Nanozyme for Detection of H_2O_2 and Evaluation of Antioxidant. *Sens. Actuators, B* **2018**, *277*, 86-94, doi:10.1016/j.snb.2018.08.097.
6. Wang, N.; Sun, J.; Chen, L.; Fan, H.; Ai, S. A $Cu_2(OH)_3Cl$-CeO_2 Nanocomposite with Peroxidase-Like Activity, and Its Application to the Determination of Hydrogen Peroxide, Glucose and Cholesterol. *Microchim. Acta* **2015**, *182*, 1733-1738, doi:10.1007/s00604-015-1506-8.
7. Socaci, C.; Pogacean, F.; Biris, A.R.; Coros, M.; Rosu, M.C.; Magerusan, L.; Katona, G.; Pruneanu, S. Graphene Oxide vs. Reduced Graphene Oxide as Carbon Support in Porphyrin Peroxidase Biomimetic Nanomaterials. *Talanta* **2016**, *148*, 511-517, doi:10.1016/j.talanta.2015.11.023.
8. Yang, H.; Zha, J.; Zhang, P.; Xiong, Y.; Su, L.; Ye, F. Sphere-Like CoS with Nanostructures as Peroxidase Mimics for Colorimetric Determination of H_2O_2 and Mercury Ions. *RSC Adv.* **2016**, *6*, 66963-66970, doi:10.1039/c6ra16619a.
9. Liu, Q.; Yang, Y.; Lv, X.; Ding, Y.; Zhang, Y.; Jing, J.; Xu, C. One-Step Synthesis of Uniform Nanoparticles of Porphyrin Functionalized Ceria with Promising Peroxidase Mimetics for H_2O_2 and Glucose Colorimetric Detection. *Sens. Actuators, B* **2017**, *240*, 726-734, doi:10.1016/j.snb.2016.09.049.
10. Chen, H.; Shi, Q.; Deng, G.; Chen, X.; Yang, Y.; Lan, W.; Hu, Y.; Zhang, L.; Xu, L.; Li, C.; et al. Rapid and Highly Sensitive Colorimetric Biosensor For the Detection of Glucose and Hydrogen Peroxide Based on Nanoporphyrin Combined with Bromine as a Peroxidase-Like Catalyst. *Sens. Actuators, B* **2021**, *343*, doi:10.1016/j.snb.2021.130104.
11. Jin, L.; Shang, L.; Guo, S.; Fang, Y.; Wen, D.; Wang, L.; Yin, J.; Dong, S. Biomolecule-Stabilized Au Nanoclusters as a Fluorescence Probe for Sensitive Detection of Glucose. *Biosens. Bioelectron.* **2011**, *26*, 1965-1969, doi:10.1016/j.bios.2010.08.019.
12. Liu, L.M.; Wen, J.; Liu, L.; He, D.; Kuang, R.Y.; Shi, T. A Mediator-Free Glucose Biosensor Based on Glucose Oxidase/Chitosan/Alpha-Zirconium Phosphate Ternary Biocomposite. *Anal. Biochem.* **2014**, *445*, 24-29, doi:10.1016/j.ab.2013.10.005.
13. Wang, L.; Zhu, W.; Lu, W.; Shi, L.; Wang, R.; Pang, R.; Cao, Y.; Wang, F.; Xu, X. One-Step Electrodeposition of AuNi Nanodendrite Arrays as Photoelectrochemical Biosensors for Glucose and Hydrogen Peroxide Detection. *Biosens. Bioelectron.* **2019**, *142*, 111577, doi:10.1016/j.bios.2019.111577.
14. Xia, Y.; Huang, W.; Zheng, J.; Niu, Z.; Li, Z. Nonenzymatic Amperometric Response of Glucose on A Nanoporous Gold Film Electrode Fabricated by A Rapid and Simple Electrochemical Method. *Biosens. Bioelectron.* **2011**, *26*, 3555-3561, doi:10.1016/j.bios.2011.01.044.
15. Aksorn, J.; Teepoo, S. Development of The Simultaneous Colorimetric Enzymatic Detection of Sucrose, Fructose and Glucose Using a Microfluidic Paper-based Analytical Device. *Talanta* **2020**, *207*, doi:10.1016/j.talanta.2019.120302.
16. Wang, K.; Li, N.; Zhang, J.; Zhang, Z.; Dang, F. Size-Selective QD@MOF Core-Shell Nanocomposites for the Highly Sensitive Monitoring of Oxidase Activities. *Biosens. Bioelectron.* **2017**, *87*, 339-344, doi:10.1016/j.bios.2016.08.026.
